# In emergency hypertension, could biomarkers change the guidelines?

**DOI:** 10.1186/s12872-024-03785-3

**Published:** 2024-03-13

**Authors:** Mohammad El Tahlawi, Scopus Mohamed Ismail, Ahmed Eldamanhory, Ayman Khorshed, Salem M. Salem

**Affiliations:** 1 Zagazig University Hospitals, Zagazig, Egypt; 2 University of Central Florida College of Medicine, Graduate Medical Education, Florida, USA; 3 Department of Laboratory Medicine and Pathology, Mayo Clinic, Rochester, MN USA

**Keywords:** Troponin, Emergency hypertension, Renal failure, Biomarker, Target organ damage, Target blood pressure

## Abstract

**Background:**

Hypertension may cause target organ damage (TOD). Target blood pressure (BP) management may not be appropriate in some conditions.

**Aim:**

We aim to assess the impact of targeted BP management in severe hypertension on renal TOD.

**Patients & methods:**

This is a prospective cohort study involving patients admitted due to severe hypertension (BP > 180/120) associated with any symptoms. The study involved patients referred to the ICU in our tertiary center during the period between August 2017 and February 2018. All patients underwent target BP treatment according to recent guidelines. Hs-Troponin T (hs-TNT) and serum creatinine (s.creat) were measured in all patients on admission and 24 h later. Patients were divided into Group A (with initial normal hs-TNT) and Group B (with initial high hs-TNT). The main outcome was in-hospital renal-related morbidity (including renal failure).

**Results:**

Four hundred seventy consecutive patients with hypertensive crises were involved in the study. Group B had a significantly higher incidence of in-hospital mortality (4 patients) and renal TOD (acute renal dysfunction) than Group A (*P* value = 0.001 and 0.000 respectively). There was a significant difference between initial s.creat on admission and follow-up s.creat values in Group B with significant elevation of their s.creat on the following 24 h (*P* = 0.002), while this difference is insignificant in Group A (*P* = 0.34). There was a significant positive correlation between hs-TNT and the follow-up s.creat (*P* = 0.004).

**Conclusion:**

In severe HTN, hs-TNT may be elevated due to marked afterload. Patients with severe HTN and high hs-TNT have higher s.creat values, which are associated with an increased risk of renal failure and in-hospital mortality if their BP decreases acutely to the guideline-target BP. Using biomarkers during the management of emergency HTN should be considered before following clinical guidelines. However, our findings do underscore the potential utility of hs-TNT as an indicator for risk stratification in patients with severe or emergency HTN.

## Key points

**Question:**

What is the relationship between troponin in emergency hypertension and the in-hospital outcome of the application of guidelines-targeted blood pressure control?

**Findings:**

In this cohort study that included 470 adults, patients with emergency hypertension with positive troponin had worse in-hospital outcomes with increased incidence of renal dysfunction if they were treated to guidelines target blood pressure.

**Meaning:**

In emergency hypertension, the management of patients should be individualized according to the results of biomarkers.

## Introduction

Hypertension (HTN) is still considered a major cause of cerebrovascular disease, with approximately 30% of adults currently diagnosed as hypertensive in the USA [1]. In addition, many hypertensive patients have uncontrolled blood pressure (BP) leading to the risk of target organ damage (TOD) [2, 3]. It is necessary to identify hypertensive patients who are at risk of developing target organ damage.

Although the diagnosis of hypertension is made when BP reaches certain thresholds, TOD due to high BP occurs on a continuous spectrum. Indeed, even early, pre-hypertensive, state may induce structural heart changes [4].

Some laboratory and electrocardiographic markers may have a role in predicting the worse prognosis of hypertension [5, 6].

Cardiac troponin I (cTnI) and T (cTnT) are the most sensitive and specific biochemical markers of myocardial cell damage [7]. They have the ability.

to detect even microscopic necrosis of myocardium [8]. These assays can detect subclinical myocardial damage in a significant proportion of patients who have no apparent cardiovascular disease [9, 10]. Thus, hs-cTNT may help identify individuals who are at risk of developing hypertensive cardiac end-organ damage, such as LVH.

Attention regarding (BP) treatment targets has been regained, and the outcome of the aggressive approach in lowering blood pressure to < 120/80 mmHg in high-risk hypertensive patients is a debatable issue [11].

The question is “Is target BP suitable for all patients? Or it should be individualized?”. Another question should be addressed; “ Is there any biomarker that could be used for guiding our goals of therapy?”

### Aim of the work

Our study aims to assess the impact of guidelines-targeted BP treatment in severe hypertension on renal TOD during the in-hospital course. In addition, we aim to find out the significance of the simple biomarker hs-TNT in guiding the management of severe HTN.

### Patients & methods

All patients admitted to our center due to severe hypertension (BP > 180/120) [10] associated with any symptoms during the period between August 2017 and February 2019 were enrolled in this study.

Patients were excluded from this study if they:

- had myocardial infarction, either STEMI or Non-STEMI;
- already had renal failure or on hemodialysis;
- had a fever or any signs of infection.
- presented with acute aortic dissection.
- presented with acute stroke or cerebral hemorrhage.
- presented with eclampsia or pre-eclampsia.

Thorough clinical examination and rapid echocardiography were done for all patients. All patients were managed according to guidelines [12–14]. Nitroglycerin infusion, due to its availability in our center, was used with titrated doses beginning with 5ugm/min increased every 3–5 min according to BP response to a maximum dose of 100ugm/min till reached the guideline target BP followed by gradual weaning. Continuous hemodynamic monitoring with careful titration according to BP response was done.

Guideline targeted BP was achieved in all patients during their hospitalization as follows: SBP has been reduced by no more than 25% within the first hour; then, if stable, to 160/100 mm Hg within the following 2 to 6 h; and then gradually to normal [12, 15].

After initiation of IV treatment, we added ACEI, ARBs, or BB according to the case scenario, as oral therapy with these agents may be very useful in malignant HTN in which the renin system is activated due to renal ischemia [15].

On admission, hs-TnT concentration was measured, using an Elecsys 2010 system (Roche Diagnostics, Basel, Switzerland), and serum creatinine (s.creat) was measured using Chemistry Analyzer AU480 (Beckman Coulter Inc., USA).

This hs-cTnT assay measures values in the range of 3 to 100,000 ng/L.

The follow-up tests were done 24 h later.

Patients were divided into 2 groups according to their initial hs-TNT values; **Group A** with initial normal hs-TNT values (< 0.003 ng/ml) and **Group B** with initial high hs-TNT values (> 0.003 ng/ml).

In-hospital morbidity and mortality were recorded for all patients.

IRB approval was obtained, and written informed consent was obtained from each patient.

### Statistics

SPSS version 2010 was used for data analysis. Continuous Variables are expressed as mean ± SD while categorical variables are expressed in frequency and percentage. Differences in the frequency of characteristics were assessed by independent sample student’s t-test for continuous variables, while Chi-square statistics were used for discrete variables. A two-tailed *P*-value, 0.05 was considered statistically significant.

Variables that had not fulfilled the normal distribution frequency conditions were treated by non-parametric tests (Mann-Witney test) or (Wilxocon test).

Logistic regression analysis was done to model the dependent and independent variables and make a univariate analysis; *P*-value of 0.05 was considered statistically significant.

## Results

**Fig. 1:**
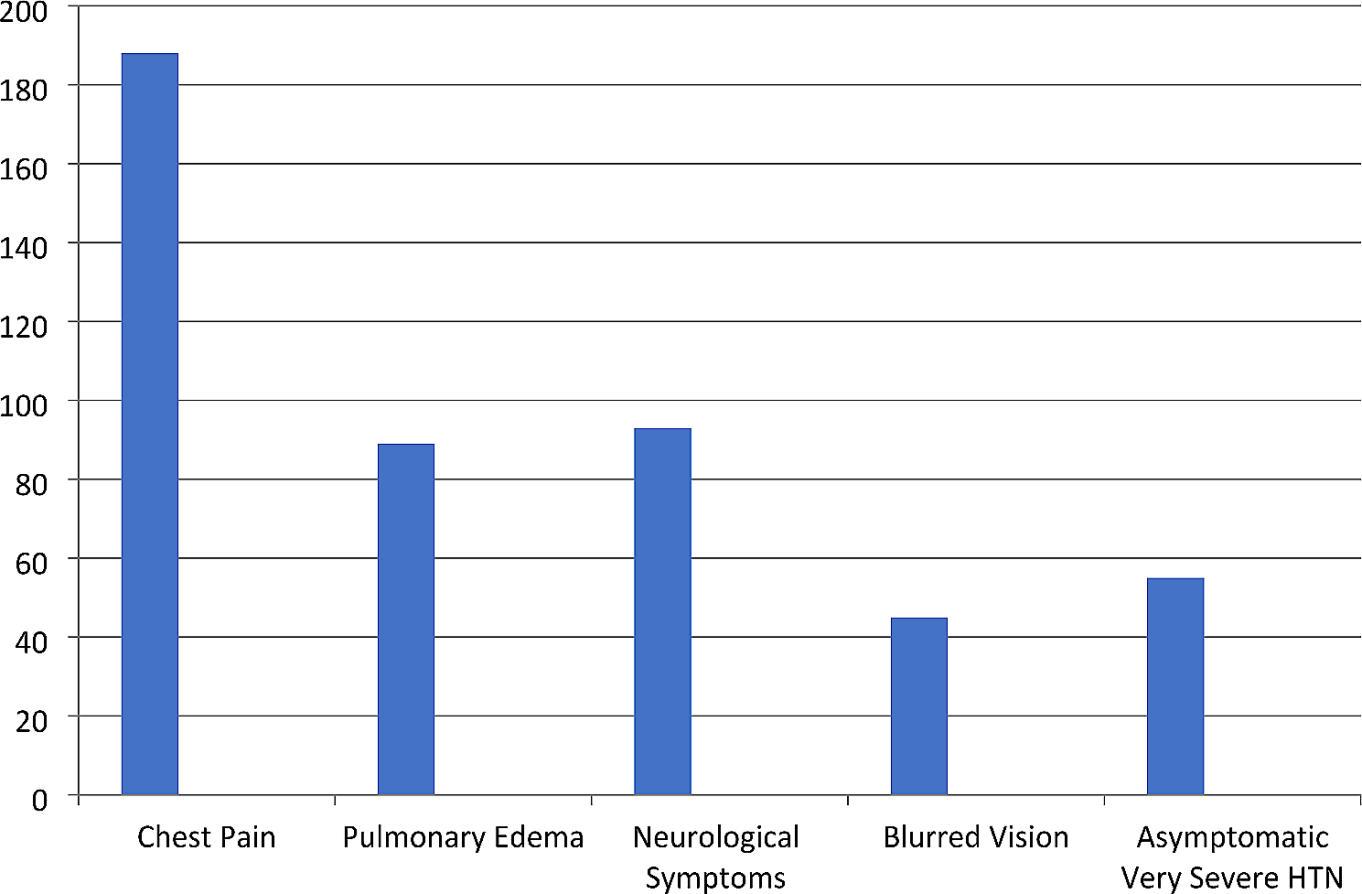
Shows the clinical presentations of patients with hypertensive crisis

Four hundred ninety-five consecutive patients with hypertensive crises with different profiles were admitted. Twenty-five patients were excluded. The remaining 470 patients were enrolled in this cohort prospective. Figure 1 shows the different symptoms associated with HTN in the study populations.

One hundred eighty patients had initial normal Hs-TNT (**Group A**) and 290 patients had initial high Hs-TNT (**Group B**).

None of the patients in either group had the history of intake of nephrotoxic drugs over the past 3 months.

### Regarding BP

Mean blood pressure measurements were (226 ± 25 for systolic, 118 ± 16 for diastolic, and 149 ± 17 for mean BP) on admission, and (157 ± 21for systolic, 92 ± 13 for diastolic, and 113 ± 16 for mean BP) 24 h after initiation of treatment. The mean duration of treatment till control of BP was 10.5 ± 5.2 h for group A and 18.7 ± 7.2 h for group B (*P* = 0.000).

### Regarding laboratory results

Hs-TnT was ≥ 0.003 ng/ml in 290 patients **(Group B)**, with mean of 0.270 ± 0.019 ng/ml (range 0.003—0.308), while **Group A** comprised 180 patients with mean hs-TNT value = 0.001 ± 0.0015 ng/ml.

Table 1 presents the difference between both groups on admission.

**Table 1:** Characteristics of patient populations on admission

Clinical factor	Group A (180) N° (%)	Group B (290) N° (%)	*P*-Value
Hypertension	74 (41%)	122 (42%)	0.7
DM	49 (27%)	64 (22%)	0.17
Age			
< 45 year	77 (43%)	104 (36%)	0.07
> 45 year	103 (57%)	185 (64%)	0.28
Smoking	112 (62%)	64 (22%)	0.01
Pre-existing PAD	25 (14%)	49 (11%)	0.18
Syst BP (mmHg)	218 ± 14	234 ± 32	0.001
Dias BP (mmHg)	114 ± 9	121 ± 20	0.001
Duration till achieving TBP (hour)	10.5 ± 5.2	18.7 ± 7.2	0.001
Creatinine (mg/dl)	1.03 ± 0.18	1.47 ± 0.12	0.01
Hs-Troponin (ng/ml)	0.001 ± 0.0015	0.270 ± 0.019	0.001

**DM**: Diabetes Mellitus, **PAD**: Periphral arterial disease, **Syst BP**: Systolic blood pressure, **Dias BP**: Diastolic blood pressure, **TBP**: Target blood pressure

In **group B**, the mean s.creat on admission was 1.47 ± 0.12 mg/dl and 2.6 ± 0.8 mg/dl 24 h later (*P* value = 0.001), while this difference was insignificant in Group A (*P* = 0.34).

There was a weak positive correlation between hs-TNT and initial (admission) s.creat (*P* = 0.06) in the whole study population but a highly significant positive correlation between hs-TNT and the follow-up (24-hour post-admission) s.creat (*P* = 0.004).

Raised initial hs-Troponin has shown to have a greater statistical power in predicting renal dysfunction in severe hypertensive patients undergoing guideline target BP control (Observed power = 0.89).

### Regarding the outcome

In **group A**, there was one death due to extensive cerebral infarction on the following day of admission, while in **group B**, there were 4 deaths due to massive cerebral hemorrhage in 2 cases and acute heart failure in the other 2 cases (diagnosed clinically and by depressed EF less than 40%) (*P* value = 0.01).

**Group B** had a significantly higher incidence of in-hospital renal dysfunction than **group A** (*P* value = 0.001) (Table 2).

**Table 2:** Difference between both groups in morbidity & mortality

Parameter	Group A N° (%)	Group B N° (%)	*P*-Value
Mortality	1 (0.5%)	4 (1.4%)	0.001
Renal Failure/Renal Dysfunction	0	153 (52.7%)	0.000
Hospital stay (Days) (mean ± SD)	1.5 ± 0.5	5.5 ± 1.5	0.000

Twenty-three patients in **group B** developed acute renal failure after control of BP to the guideline level, while 130 cases developed moderate renal dysfunction in the form of rising s.creat and reduction of eGFR during in-hospital stay.

The multivariate analysis shows that hs-TnT is still the only independent predictor of renal dysfunction in severe hypertensive patients undergoing guideline target BP control, even after controlling for smoking, systolic blood pressure, diastolic blood pressure, and initial s.creatinine. The coefficients for smoking, systolic BP, diastolic BP, and initial s.creatinine indicate their respective contributions to the likelihood of renal dysfunction after controlling for the other variables in the model (Table 3).

**Table 3:** Multivariate analysis for predictors of renal dysfunction in severe hypertensive patients undergoing guideline target BP control

Variable	Coefficient	Standard Error	t-value	*P*-value
Intercept	1.234	0.567	2.179	0.031
hs-TnT (ng/L)	0.567	0.123	4.605	< 0.001
Smoking (Yes/No)	-0.123	0.045	-2.734	0.006
Systolic BP (mmHg)	0.045	0.012	3.653	< 0.001
Diastolic BP (mmHg)	0.056	0.013	4.324	< 0.001
Initial s.creatinine (mg/dL)	0.234	0.056	4.856	< 0.001

The regression analysis shows that hs-TnT is the only independent predictor of renal dysfunction in severe hypertensive patients undergoing guideline target BP control (Table 4).

**Table 4:** Regression analysis for independent predictors of renal dysfunction in severe hypertensive patients undergoing guideline target BP control

Variable	Coefficient	Standard Error	t-value	*P*-value
Intercept	1.234	0.567	2.179	0.031
hs-TnT (ng/L)	0.567	0.123	4.605	< 0.001

## Discussion

In our cohort, we studied the impact of guidelines-targeted BP control in patients with emergency HTN on their renal outcome. We found that patients with positive hs-troponin on admission have worse outcomes regarding renal dysfunction as well as mortality.

Resultant pressure overload due to severe HTN may result in subclinical myocardial wall remodeling and potential ischemic cardiomyocyte injury [16].

Severe hypertension exerts very high-pressure overload that causes relative ischemia and necrosis or minute damage to myocytes, and thus troponin release. This effect similarly occurs in any other cause of increased pressure loads [17, 18]. In addition, activation of the sympatho-adrenal system in hypertensive patients leads to increased catecholamine release and subsequent myocyte injury [19].

Optimal SBP targets from ACCORD [20], INVEST [21], and ONTARGET [14] seem to be between 120 and 140 mmHg. A comprehensive meta-analysis [22] of 17 trials including SPRINT, aiming at assessing optimal BP targets, found that the balanced efficacy and safety could be achieved at 130 mmHg.

More consideration was given to the results from SPRINT, suggesting reduced CV risk with SBP treatment target at 120 mmHg [23].

The concept of “the lower the better” in case of hypertension in diabetic, renal, or CAD patients should be revised. The guidelines-targeted BP reduction in case of severe hypertension could be changed if we found an increased risk of morbidity and mortality by applying this strategy.

In the current study, we found that emergency hypertensive patients with initially raised hs-TNT had higher in-hospital mortality and morbidity (renal dysfunction) than those with normal hs-TNT when we applied the guidelines in controlling their BP. They developed deterioration of their renal function if their BP was controlled to the guideline level. In addition, the degree of deterioration of s.creat was positively correlated with the initial level of hs-TNT. However, those with initial normal hs-TNT levels would get benefit of guideline-targeted BP control.

Hypertension-induced TOD may affect the kidneys in its early stages. The risk of albuminuria progression was assessed in meta-analysis and found to be significantly reduced after intensive BP control [24].

Another study found that greater proteinuria and reduced eGFR in the general population are associated with masked hypertension [25]. This may suggest that the damaging effect of hypertension might affect the kidney earlier than other organs, causing TOD for the kidney even before the diagnosis of HTN or the occurrence of any symptoms.

In our study, there was a significant difference between both groups with and without elevated hs-TNT regarding initial s-creat, with higher values in group B with elevated initial hs-TNT. This may indicate that the kidney has been already affected by HTN in those patients to a subclinical degree. Therefore, the relatively marked reduction of BP (according to the guidelines target) may disturb the renal autoregulation that seems to be developed with time in those patients.

Our conclusion was supported by another study conducted by Seccia et al. They found that a relatively high proportion of hypertensive patients develop mild-to-moderate hypertensive nephrosclerosis. This pathology was found to progress into end-stage renal disease only in a relatively small percentage, especially those with prolonged uncontrolled SBP [26].

In this context, a more personalized, biomarker-guided, approach to BP treatment in this group with severe HTN may be a preferred alternative.

We hypothesize that target BP should not be a solid figure and should be individualized. It depends on the degree of TOD. Elevated hs-TNT, as an indicator of TOD, could be used to guide BP control. When hs-TNT is not elevated, intense BP control to the guideline-targeted BP could be applied. However, if hs-TNT is elevated during the sitting of severe HTN, more caution should be exerted not to reduce BP markedly or over a short time. In this condition, the reduction of BP just to the level making the hs-TNT value return to normal seems to be appropriate in the first days. Longer duration may be better needed before returning the BP to normal.

Patients with positive hs-troponin on admission may benefit from closer monitoring, cautious blood pressure control over a longer period and potentially more frequent assessment of renal function. These individuals may be at higher risk for renal dysfunction and mortality, so tailoring their treatment approach to address these risks could be beneficial. A multidisciplinary approach involving cardiologists, nephrologists, and intensivists may be considered to optimize the management of these high-risk patients.

In a study done on isolated systolic hypertension in older adults [27], the authors concluded that cardiac biomarkers hs-cTnT and NT-proBNP might be used to identify older adults who would benefit from more intensive BP therapy to reduce systolic hypertension. They thought that high biomarker levels might identify older patients who could benefit from intensive BP treatment. This conclusion may conflict with our findings that intense BP control increases the risk of mortality and renal failure in patients with high hs-TNT. This controversy may be due to the difference in studied populations. Those in our study had severe or emergency HTN, which might differ in their myocardial tolerance to pressure overload and in TOD risk at lower cTnT thresholds.

Cardiac troponins may be elevated in asymptomatic end-stage renal patients [28]. The mechanisms causing such biomarker elevation in renal patients are not clear. There is emerging evidence that troponin elevation in asymptomatic renal patients indicates subclinical myocardial necrosis or minute injury [29].

In a trial aiming to determine the independent risk factors of CKD progression, higher hs-TNT was significantly associated with 1.5-fold of the composite outcome of CKD deterioration. This association was similar to or stronger than that of high systolic BP [30].

The association between cardiac biomarkers such as hs-TNT and NT-proBNP and CKD may indicate their value as markers of cardiorenal syndrome [30, 31].

None of our patients took nephrotoxic drugs over the past 3 months prior to admission. While certain medications, such as ACE inhibitors and angiotensin receptor blockers, can decrease GFR by impacting renal hemodynamics. Drugs that affect kidney perfusion and haemodynamics may safeguard nephrons against hyperfiltration, which can lead to CKD progression [32].

The decrease in all-cause mortality with more intensive blood pressure lowering was also demonstrated in the SPRINT trial; however, in the subgroup of chronic kidney disease, there was not any significant reduction in risk for the primary composite endpoint of cardiovascular morbidity and mortality in those treated with more intensive therapy [33]. Post hoc analysis of some studies demonstrated higher mortality associated with more systolic hypotension [34].

## Conclusion

In severe HTN, hs-TNT may be elevated due to marked afterload. Patients with severe HTN and high hs-TNT have higher s.creat values, which are associated with an increased risk of renal failure and in-hospital mortality if their BP decreases acutely to the guideline-target BP. Using biomarkers during the management of emergency HTN should be considered before following clinical guidelines. However, our findings do underscore the potential utility of hs-TNT as an indicator for risk stratification in patients with severe or emergency HTN. Further research with larger and more diverse cohorts is needed to fully assess the role of biomarkers in guiding the management of hypertensive emergencies Hs-TNT should be considered as an indicator to guide the strategy of managing of patients with severe or emergency HTN.

### Take-Home message

In the context of severe HTN, biomarker target BP control should be applied instead of guideline target BP control. When hs-TNT and s.creat are elevated, BP should not be markedly reduced, otherwise, renal failure may develop. Nevertheless, BP should be controlled just to the level that makes the hs-TNT value return to normal. However, in patients with normal hs-TNT values, BP could be controlled to the guideline target.

Therefore, target BP should be individualized instead of being a universal value.

### Limitations

The previous data on renal function before admission was not available. The study involved a single tertiary center.

## Data Availability

The datasets used and/or analysed during the current study are available from the corresponding author on reasonable request.
